# Gut microbes participate in food preference alterations during obesity

**DOI:** 10.1080/19490976.2021.1959242

**Published:** 2021-08-23

**Authors:** Alice de Wouters d’Oplinter, Marialetizia Rastelli, Matthias Van Hul, Nathalie M. Delzenne, Patrice D. Cani, Amandine Everard

**Affiliations:** a Metabolism and Nutrition Research Group, Louvain Drug Research Institute, Walloon Excellence in Life Sciences and BIOtechnology (WELBIO), UCLouvain, Université Catholique De Louvain, Brussels, Belgium; b Metabolism and Nutrition Research Group, Louvain Drug Research Institute, UCLouvain, Université Catholique De Louvain, Brussels, Belgium

**Keywords:** Gut microbiota, reward, hedonic, food intake, obesity, fecal material transplantation

## Abstract

Hypothalamic regulations of food intake are altered during obesity. The dopaminergic mesocorticolimbic system, responsible for the hedonic response to food intake, is also affected. Gut microbes are other key players involved in obesity. Therefore, we investigated whether the gut microbiota plays a causal role in hedonic food intake alterations contributing to obesity. We transferred fecal material from lean or diet-induced obese mice into recipient mice and evaluated the hedonic food intake using a food preference test comparing the intake of control and palatable diets (HFHS, High-Fat High-Sucrose) in donor and recipient mice. Obese mice ate 58% less HFHS during the food preference test (*p* < 0.0001) than the lean donors, suggesting a dysregulation of the hedonic food intake during obesity. Strikingly, the reduction of the pleasure induced by eating during obesity was transferable through gut microbiota transplantation since obese gut microbiota recipient mice exhibited similar reduction in HFHS intake during the food preference test (40% reduction as compared to lean gut microbiota recipient mice, *p* < 0.01). This effect was associated with a consistent trend in modifications of dopaminergic markers expression in the striatum. We also pinpointed a highly positive correlation between HFHS intake and *Parabacteroides* (*p* < 0.0001), which could represent a potential actor involved in hedonic feeding probably through the gut-to-brain axis. We further demonstrated the key roles played by gut microbes in this paradigm since depletion of gut microbiota using broad-spectrum antibiotics also altered HFHS intake during food preference test in lean mice. In conclusion, we discovered that gut microbes regulate hedonic aspects of food intake. Our data demonstrate that gut microbiota modifications associated with obesity participate in dysregulations of the reward and hedonic components of the food intake. These data provide evidence that gut microbes could be an interesting therapeutic target to tackle hedonic disorders related to obesity.

## Introduction

In the physiopathology of obesity, overeating and consumption of calorie-dense food are major aspects contributing to a positive energy balance (energy input is greater than energy output) and the storage of fat.^1^ In this context, the reward system, that drives eating behaviors associated with pleasure, is over-stimulated and becomes the major driver for food intake.^1–4^ Palatable food, rich in fat and sugar, can stimulate dopaminergic neurons and induce a release of dopamine mainly in the cortico-limbic areas of the brain (including the striatum, nucleus accumbens and prefrontal cortex).^5–7^However, obesity, which is often the result of long-term overeating, is associated with a reduction of dopamine concentration in response to palatable food intake and a downregulation of dopaminergic markers. The expressions of dopamine receptors 1 (D1R) and 2 (D2R) are decreased, as well as the rate-limiting synthesizing enzyme (tyrosine hydroxylase, TH), whereas the dopamine transporter (DAT) is increased.^8–12^ This hypofunctioning of the dopamine pathway has been suggested to feed the vicious circle of weight gain since it leads to an increase in the meal size of fatty and sweet food in an attempt to feel the same rewarding effect as before the development of obesity.^13^ The gut microbiota plays a key role in the gut-to-brain axis influencing the food behavior. For instance, gut microbes have been shown to modulate the production of satiety hormones (glucagon-like peptide-1 (GLP-1) and peptide YY (PYY)), neurotransmitters (including GABA (γ-aminobutyric acid), dopamine, serotonin) and to interact with the vagus nerve.^14,15^ Mechanisms underlying the regulation of homeostatic food intake through the gut-to-brain axis are well-studied and supported by strong literature consistencies.^1^ In contrast, mechanisms of hedonic food intake have often been overlooked. It has only been a decade since de Araujo et al. demonstrated that post-ingestive signals, different from oral taste perception, were involved in triggering dopamine release in cortico-limbic areas of the brain.^16^ These findings strengthened the gut as a key sensor in the regulation of food intake. However, so far, nothing has been shown about the direct role of the gut microbiota on the hedonic food intake. Therefore, we aimed to demonstrate the causal role of the gut microbiota in the hedonic responses to palatable food by using fecal microbiota transplantation and subsequent appropriate food behavioral tests.

## Results

### DIO donor mice show alterations in hedonic eating

**Figure 1.**
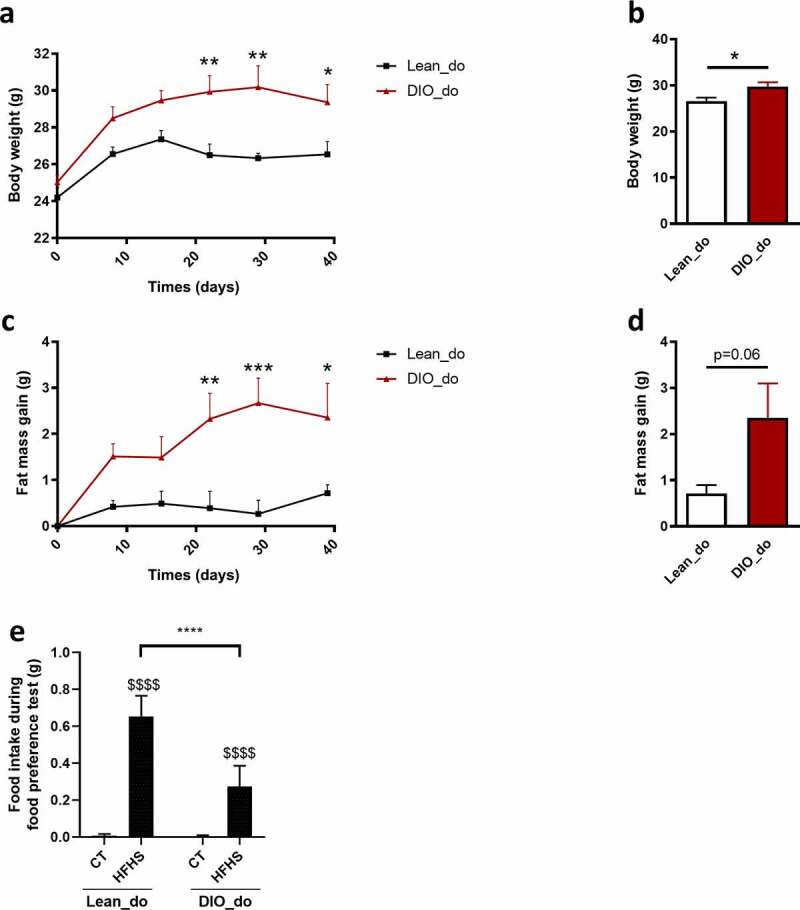
Obese mice present a reduced food preference for HFHS compared to lean mice

First, we exposed 10 donor mice to a low-fat (control, CT) or high-fat diet (HFD) for 5 weeks to induce a lean or obese phenotype (diet-induced obesity, DIO), respectively. As expected, mice fed with a HFD showed an increase of 12% in body weight (Figure 1a-b) and 230% in fat mass gain (Figure 1c-d) compared to CT-fed mice. Then, in order to study the hedonic component of food intake, we analyzed the pleasure associated with palatable food consumption in these mice.

**(a,b)** Body weight evolution (g) **(a)** and final body weight (g) **(b)** of lean (Lean_do) and DIO donor mice (DIO_do) after a 5 weeks period. **(c,d)** Fat mass gain evolution (g) **(c)** and final fat mass gain (g) **(d)** of lean (Lean_do) and DIO donor mice (DIO_do). **(e)** Food preference test showing total HFHS and CT intake after 180 minutes of test by lean (Lean_do) and DIO donor mice (DIO_do). Data are shown as mean ± SEM (n = 5/group). *P*-values were obtained after Two-way ANOVA, followed by Bonferroni post-hoc test **(a, c, e**), unpaired Student’s t-test **(b,d)**. *: *p*-value ≤0,05; **: *p*-value ≤ 0,01; ***: *p*-value ≤ 0,001; ****: *p*-value ≤ 0,0001. :$$$$: p-value≤ 0.0001 between CT vs HFHS intake (e). $$$$: *p*-value ≤ 0.0001 between CT vs HFHS intake **(f)**.

To assess spontaneous hedonic food intake, the donor mice underwent a food preference test in which they were exposed for the first time to a palatable diet (High-Fat High-Sucrose, HFHS). During this food preference test, donor mice were exposed to HFHS and low-fat control diet (CT) for 3 hours during the light phase, and we recorded the consumption of each diet (Figure 1e and Fig. S1). Both lean and obese mice preferred the HFHS diet to CT as they ate more HFHS than CT during the food preference test. However, lean mice showed a faster tropism toward HFHS since they ate significantly more HFHS than CT from the beginning of the test, whereas DIO mice preferred a significantly palatable diet to control diet only after 90 min (Fig. S1). Overall, DIO mice were significantly less attracted to palatable diet, eating 58% less HFHS (*p* < 0.0001) than lean mice over the whole food preference test (Figure 1e).

### Obese gut microbiota transplantation transfers alterations in hedonic eating associated with obesity

**Figure 2.**
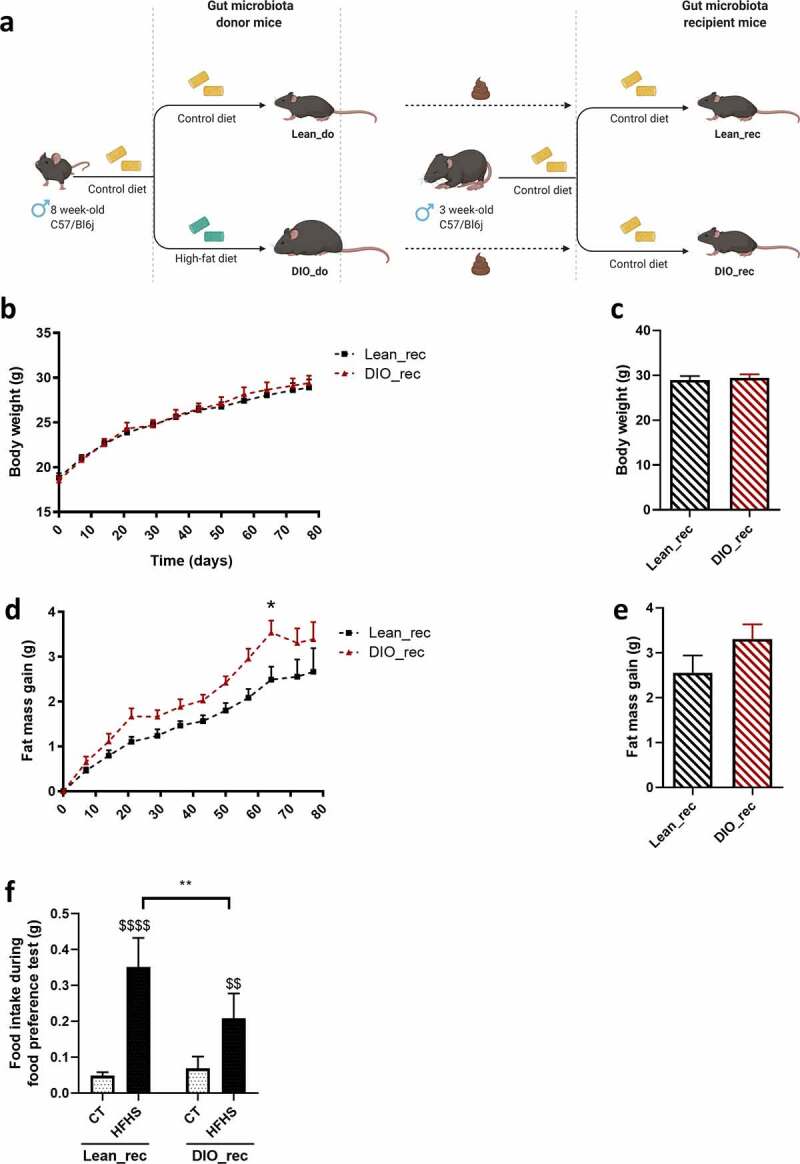
Recipient mice show hedonic food behavior similar to donor mice after fecal transplantation

To study the causal role of the gut microbiota in obesity-related hedonic eating disorders, we transplanted the gut microbiota from two lean and two obese donor mice into seven and eight recipient mice, respectively. All recipient mice were fed with the same low-fat, control diet during the whole experiment (Figure 2a).

**(a)** Experimental plan of the FMT protocol. **(b,c)** Body weight evolution (g) **(b)** and final body weight (g) **(c)** of lean (Lean_rec) and DIO recipient mice (DIO_rec). **(d,e)** Fat mass gain evolution (g) **(d)** and final fat mass gain (g) **(e)** of lean (Lean_rec) and DIO recipient mice (DIO_rec). **(f)** Food preference test showing total HFHS and CT intake after 180 minutes of test by lean (Lean_rec) and DIO recipient mice (DIO_rec). Data are shown as mean ± SEM (n = 7–8/group). *P*-values were obtained after Two-way ANOVA, followed by Bonferroni post-hoc test **(b,d,f)** or unpaired Student's t-test **(c,e)**. *: *p*-value ≤ 0,05; **: *p*-value ≤ 0,01. $$: *p*-value < 0.01; : *p*-value ≤ 0.0001 between CT vs HFHS intake **(f)**.

Lean and obese gut microbiota recipient mice (Lean_rec and DIO_rec, respectively) did not show any difference in terms of body weight (Figure 2b-c) or fat mass gain (Figure 2d-e). However, DIO gut microbiota recipient mice tended to gain more fat mass over time, with a statistical significance at day 64 (Figure 2d). In order to investigate the energy metabolism of lean and obese gut microbiota recipient mice, we also performed precise measurements of O_2_ consumption and CO_2_ production in metabolic chambers. We did not observed any differences between mice receiving an obese or lean gut microbiota (Fig. S2). These results suggest that donor mice did not transfer their obese phenotype to recipient mice in terms of fat mass and body weight after fecal transplantation. It is worth noting that the transfer of the obese phenotype after fecal material transplantation has been debated in the literature.^17^

Interestingly, during the entire follow-up, lean and obese gut microbiota recipient mice had a similar intake of control diet (Fig. S3a-b). However, during their first exposure to palatable food (*i.e*. food preference test), we revealed differences in HFHS intake (Figure 2f and Fig. S3c). Lean gut microbiota recipient mice displayed a faster preference for HFHS than DIO gut microbiota recipient mice. In fact, lean gut microbiota recipient mice ate significantly more HFHS than CT diet after 90 minutes of test, whereas the difference between HFHS and CT intake in DIO gut microbiota recipient mice was only significant after 150 and 180 min (Fig. S3c). Like the donor mice, the two recipient groups showed a preference for a palatable diet over a CT diet. Strikingly, the total HFHS intake was 40% less important in DIO gut microbiota recipient mice compared to lean gut microbiota recipient mice (*p* < 0.01, Figure 2f). These results demonstrate that lean and DIO gut microbiota recipient mice show similar patterns in terms of hedonic eating behavior as their respective microbiota donors and this effect is independent of obesity development or non-hedonic feeding behavior. Of note, ambulatory activity during the test was comparable between recipient mice, suggesting a similar exploratory behavior toward this novel food high in sugar and fat (Fig. S3d). Taken together, we uncovered a causal role of the gut microbiota in the hedonic food behavior alterations associated with obesity.

### Dopaminergic markers in the striatum suggest a hypofunctional food reward system in DIO recipient mice

Pleasure associated with palatable food intake is mainly driven by dopaminergic pathways in the mesocorticolimbic system. Indeed, ingestion of a diet rich in fat and sugar has been shown to be associated with the release of dopamine in the striatum in proportion to the self-reported level of pleasure derived from eating the food.^18^ Dopamine receptors 1 and 2 (D1R and D2R) are the most expressed dopamine receptors of the reward system,^19^ and the scientific literature describes a downregulation of these receptors in the context of obesity in humans and rodents, which in turn is associated with a reduction of the pleasure related to palatable food ingestion.^8,11^ Since transplantation of obese gut microbiota replicated food preference alterations associated with obesity (Figure 2f), we wondered if this was associated with modifications in dopaminergic markers. Therefore, we investigated the expression of dopaminergic markers in the striatum of recipient mice by qPCR.

**Figure 3.**
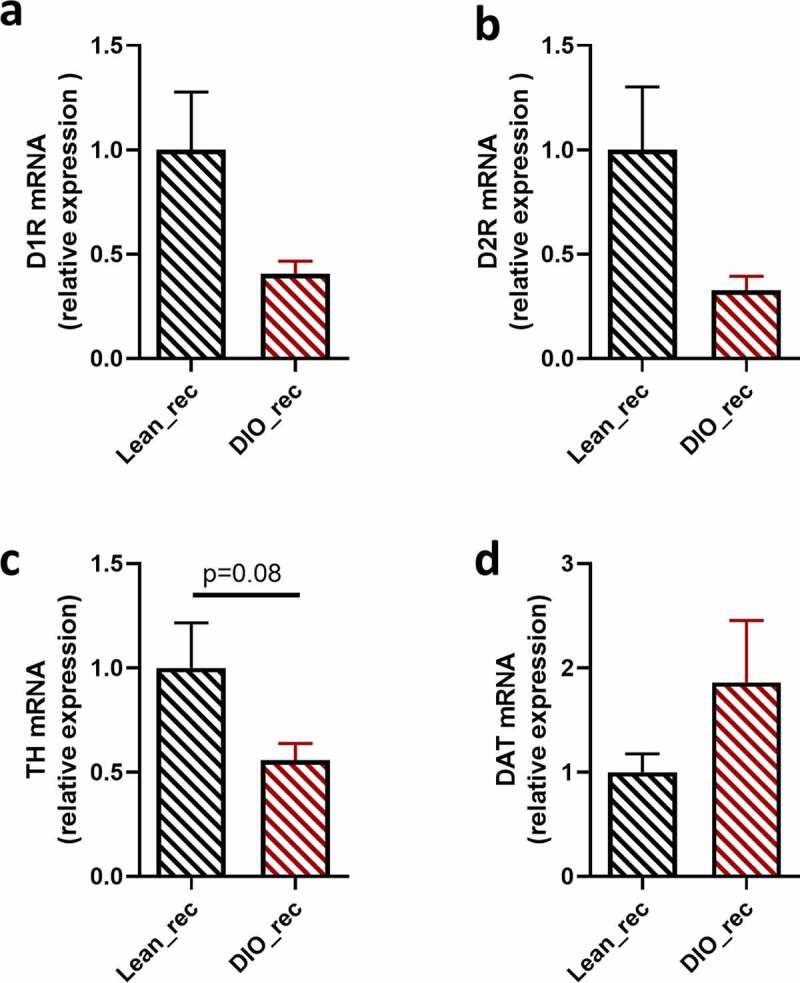
Alterations in dopaminergic signaling in recipient mice with obese gut microbiota

Our results show that after microbiota transplantation, DIO recipient mice express at least 60% less D1R and D2R in the striatum compared to lean recipient mice, although this failed to pass the statistical threshold due to high variability in the Lean_rec group (*p* > 0.05, Figure 3a-b). The expression of tyrosine hydroxylase (TH), the rate-limiting enzyme synthesizing dopamine, was also decreased (50%) in mice receiving obese microbiota compared to mice receiving lean microbiota (*p* > 0.05, Figure 3c). In line with these results, the dopamine transporter (DAT), responsible for the recapture of around 80% of the dopamine released, was two-fold more expressed in DIO_rec compared to Lean_rec (*p* > 0.05, Figure 3d), suggesting low function of the dopaminergic system in obese gut microbiota transplanted mice. Of note, the modifications of expression of dopaminergic markers are not associated with changes in the ambulatory activity (Fig. S2 a-b and Fig. S3d) suggesting that the qPCR results observed in the striatum are specific to the reward system rather than the motor function.

Striatal mRNA expressions of dopamine receptor 1 (D1R) **(a)**, dopamine receptor 2 (D2R) **(b)**, tyrosine hydroxylase (TH) **(c)** and dopamine transporter (DAT) **(d)** were measured by real-time qPCR in lean (Lean_rec) and DIO recipient mice (DIO_rec). Data are shown as mean ± SEM (n = 7–8/group). *P*-values were obtained after unpaired Student’s t-test **(c)** or non-parametric Mann–Whitney test **(a,b,d)**.

Besides the dopaminergic system in the striatum, other brain areas are involved in food reward such as caudate putamen, nucleus accumbens, and prefrontal cortex. Therefore, we further investigated and analyzed mRNA levels of the dopaminergic markers in these regions (Table S1). We did not observe any difference between lean and obese gut microbiota recipient mice in these brain areas, suggesting that modulations of the expression of dopaminergic markers between lean and obese gut microbiota recipient mice seem to be specific to the striatum.

### Fecal material transplantation from obese donors into lean recipient mice is efficient

**Figure 4.**
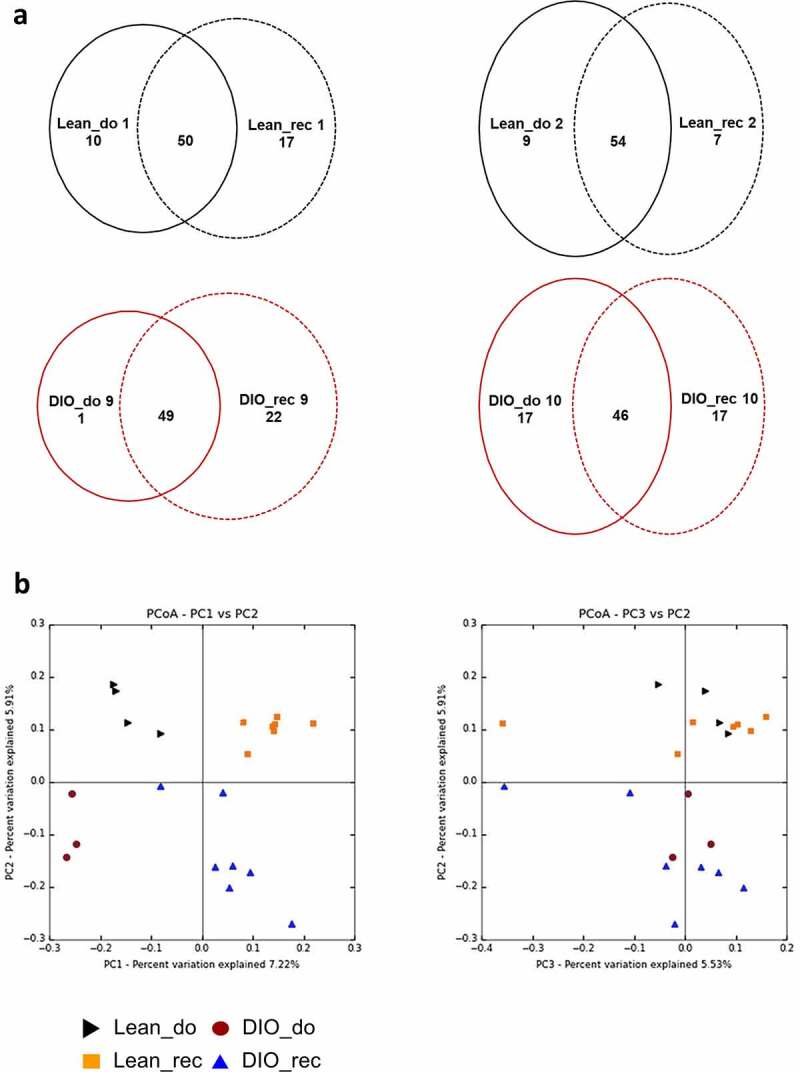
The gut microbiota of recipient mice is similar to the gut microbiota from donor mice

To validate the efficiency of the gut microbiota transplantation, bacterial composition of cecum contents from donor and recipient mice were analyzed using 16S rRNA sequencing. We compared common OTUs (Operational Taxonomic Units) between donors and recipients at the end of each experiment, just after food preference tests (Figure 4a). Two mice from each donor group (CT-fed or HFD-fed) were donors for 7 Lean_rec and 8 DIO_rec recipient mice, respectively, with one donor mouse for 3 or 4 recipient mice. Venn diagram showed a high similarity of OTUs (more than 50%) between donors and recipients, confirming the colonization of antibiotic-treated recipients with gut microbiota from donors (Figure 4a).

Venn diagram is based on OTUs similarity between donor (Lean_do and DIO_do) and recipient (Lean_rec and DIO_rec) mice **(a)**. Principal coordinates analysis (PCoA) is based on the unweighted UniFrac analysis on operational taxonomic units (OTUs). Each symbol representing a single sample is colored according to its group (**b).**

Furthermore, as represented on the PCoA, obese donors, and obese gut recipient mice have gut microbiota profiles that differ from lean donors and lean gut microbiota recipient mice according to the principal component PC2 (Figure 4b).

### Parabacteroides represent a potential link in the gut-to-brain axis controlling hedonic food intake

**Figure 5.**
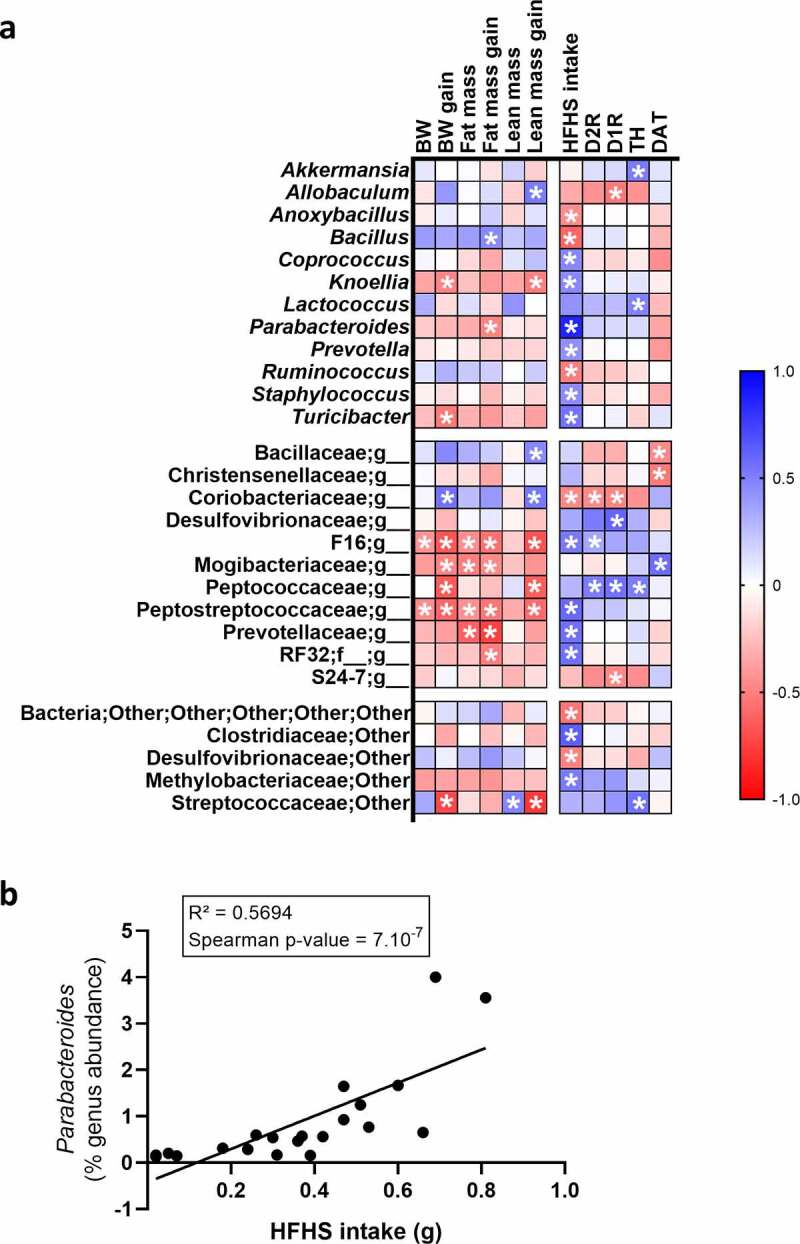
Correlations between gut microbes and dopaminergic markers

As a preliminary approach to highlight a potential link between the gut microbiota and the food reward system in the context of obesity, we used Spearman’s correlations to establish associations between several parameters of the food reward system and the gut microbiota. Data from donor and recipient mice were combined to create the correlation matrix. The heatmap showed that 18 OTUs correlated with the total HFHS intake measured during the food preference test (Figure 5a). In addition, positive correlations were found between the unidentified genus of the Peptococcaceae family and the mRNA expression of D1R, D2R, and TH (Figure 5a). However, after correcting for multiple comparisons using the FDR (false discovery rate) method, only *Parabacteroides* remained highly positively correlated with the HFHS intake (Figure 5b). This suggested that the more *Parabacteroides* the mice had, the more HFHS they ate during the food preference test. Based on this, *Parabacteroides* could represent a potential link between the gut microbiota and hedonic food behavior.

Heatmaps of bacterial composition and food reward patterns. Spearman’s correlations were calculated for each parameter for donor and recipient mice **(a)**. *P*-values were obtained after Spearman’s correlation test. *: p ≤ 0,05; Spearman’s correlation after FDR correction **(b)**.

### Depletion in gut microbiota over-stimulates hedonic eating in lean mice

**Figure 6.**
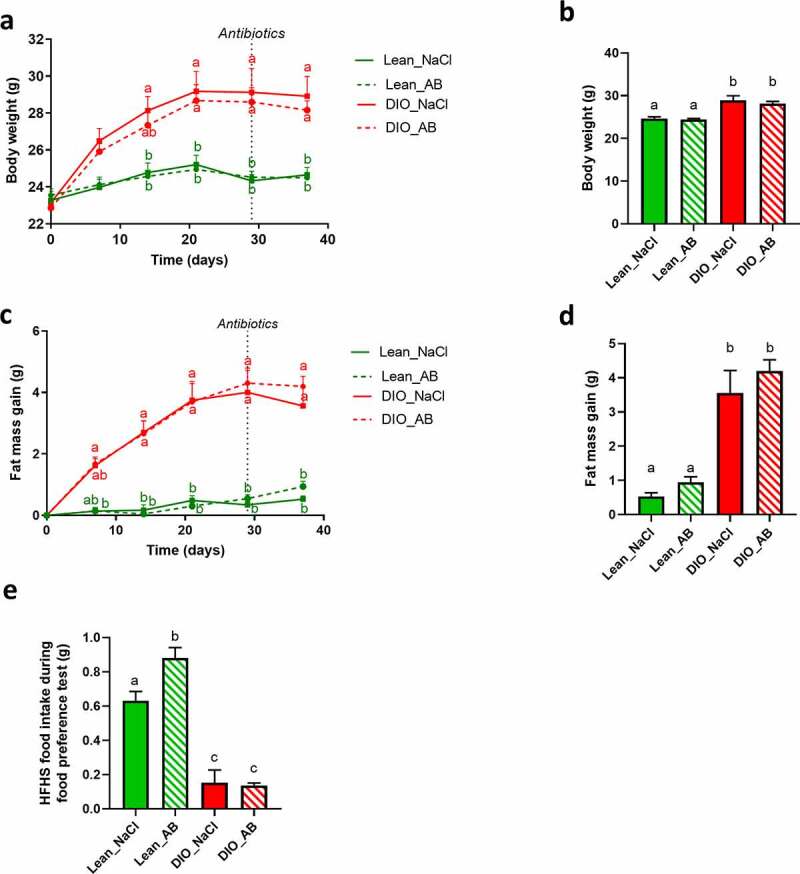
Antibiotic treatment affects food preference and HFHS intake independently of body weight and fat mass accumulation

To further investigate the causal role of the gut microbiota in the food reward system, we depleted the gut microbiota of mice by using a mixture of antibiotics. For this purpose, another cohort of mice, including mice under CT (Lean) and HFD (DIO) was used and followed during 5 weeks. Within each group, half of the mice received daily a mixture of antibiotics by oral gavage (Lean_AB and DIO_AB). A saline solution was administered daily by oral gavage to the other half (Lean_NaCl and DIO_NaCl). We first validated the model of depletion of the gut microbiota by quantifying the total bacterial load by qPCR (Fig. S4a). After 5 days of daily gavage with antibiotics, 99% of the total bacterial load was suppressed in each group. As predicted, mice under HFD gained significantly more weight (Figure 6a-b and had a higher fat mass gain than the CT-fed mice (Figure 6c-d). Importantly, antibiotic treatment did not affect body weight (Figure 6a-b) or fat mass gain (Figure 6c-d).

Body weight evolution **(a)** and final body weight **(b)** of lean and DIO mice treated with antibiotics (Lean_AB and DIO_AB) or saline (Lean_NaCl and DIO_NaCl). Fat mass gain evolution **(c)** and final fat mass gain **(d)** of lean and DIO mice treated with antibiotics (Lean_AB and DIO_AB) or saline (Lean_NaCl and DIO_NaCl). **(e)** Food preference test showing total HFHS and CT intake after 180 minutes of test by lean and DIO mice treated with antibiotics (Lean_AB and DIO_AB) or saline (Lean_NaCl and DIO_NaCl). Data are shown as mean ± SEM (n = 10/group). *P*-values were obtained after Two-way ANOVA, followed by Bonferroni post-hoc test **(a,b,c,d,e)**. Different superscript letters represent significant *p*-values between groups and type of diet (CT or HFHS) **(e)**.

To investigate the impact of the depletion of the gut microbiota on the “liking” component of the reward (pleasure), a food preference test was done in this cohort (Figure 6e and Fig. S4b). Obese mice (DIO_NaCl) ate 76% less HFHS than lean mice (Lean_NaCl) during the food preference test (*p* < 0.0001), as already observed in our first cohort of mice. These results demonstrated the reproducibility of our data and thereby their validity. We also observed that even after treatment with antibiotics, DIO mice (DIO_AB) displayed the same behavior and consumed 84% less HFHS compared to lean mice supplemented with antibiotics (Lean_AB) (*p* < 0.0001). Strikingly, this test showed that lean mice treated with antibiotics (Lean_AB) ate significantly more HFHS than lean mice with an intact gut microbiota (Lean_NaCl) from the first hour until the end of the test (Figure 6e and S4b). These results demonstrate for the first time that a healthy gut microbiota is involved in hedonic response to palatable food and that any type of alteration of the gut microbiota composition might affect the rewarding response to food.

## Discussion

In this study, we provided data supporting the dysregulation of the reward system associated with obesity from a food behavioral point of view.^20,21^ Indeed, during the food preference test, obese mice showed a delayed preference for HFHS and ate significantly less palatable food compared to lean mice. These results suggest that eating a high-fat diet during a long period of time results in a hypofunctioning of the food reward system: palatable food is attributed to less hedonic value in obese mice. In the mesocorticolimbic system, this implies a downregulation of the dopaminergic system associated with weight and fat mass gain as seen in the literature.^8^ In this study, we focused on the “liking” component of the food reward, associated with the pleasure induced by palatable food. However, food reward also includes two other components, not investigated in the present study, such as the “wanting” (the motivation to obtain a reward) and the “learning” (the capacity to associate an environment with pleasure related to palatable food intake). These two other components can be evaluated with behavioral tests such as the operant wall and the conditioned place preference, respectively.

Importantly, by using two different approaches we discovered that the gut microbiota is an active regulator of the hedonic component of the food intake. Indeed, we demonstrated that transplanting the gut microbiota from obese donors into lean recipient mice replicated the obese altered hedonic behavior. In addition, we showed that the depletion of the gut microbiota using an antibiotic treatment increased the consumption of palatable food during a food preference test. Altogether, we found out that alterations of the food intake related to the pleasure during obesity was at least partially explained by gut microbiota modifications associated with obesity, but not due to obesity itself. Indeed, the altered hedonic behavior of obese mice was transferable into recipient mice through gut microbiota transplantation, but this was independent of obesity development and HFD consumption since obese gut microbiota recipient mice displayed the same body weight and similar energy metabolism as lean gut microbiota recipient mice. There was a minor increase in fat mass gain in obese gut microbiota recipient mice as compared to lean gut microbiota recipient mice, but this had negligible effects on body weight evolution. Even though some data in the literature suggest that obese phenotype can be transferred by the gut microbiota, this hypothesis has been debated since the type of diet plays a key role in the development of fat mass gain and obesity.^17^ Importantly, microbiota analysis revealed a high similarity between gut bacterial composition from donor and recipient mice, suggesting that the gut microbiota inoculum from donor efficiently engrafted the recipient cecum.

Importantly, a key question remains a matter of debate in the scientific literature as discussed by Berthoud et al; it is not clear whether predisposing differences in reward functions cause overeating and weight gain, or whether repeated exposure or secondary effects of the obese state alter reward functions.^22^ Using caloric restriction experiments in obese rodents, they demonstrate that weight loss resulted in a restoration of the sucrose concentration–response curve similar to the curve observed in lean rats. They concluded that most of the difference between lean and obese rats was due to secondary effects of the obese state, not to preexisting differences in reward processing. Using obese gut microbiota transplantation, our data demonstrate that an altered gut microbiota consequently to obesity contributes to alterations in hedonic food intake. Thereby, we provide new insight into the role of obesity state in hedonic alterations suggested by Berthoud.

In addition to behavioral evidence of a dysregulation of the food reward system in obese gut microbiota recipient mice, qPCR analysis also suggested a hypofunctioning of the dopaminergic pathways at the level of the striatum as typically described during obesity. All the dopaminergic markers investigated for synthesis and receptors are decreased, whereas the dopamine transporter, responsible for dopamine recapture, is increased in DIO_rec compared to Lean_rec mice. Although the striatum is described as the main structure involved in the gut-induced reward,^23^ other structures are also involved in the food reward system. These include the nucleus accumbens, the prefrontal cortex, and the caudate putamen. However, we did not observe any difference in the expression of dopaminergic markers in these brain areas. Hence, our data suggest that modulations of the expression of dopaminergic markers between lean and obese gut microbiota recipient mice are specific to the striatum. Besides the dopaminergic system, other systems are involved in the food reward. These include opioids such as dynorphin that have been identified as key mediators involved in the pleasure induced by palatable food and could represent another interesting perspective to investigate in this context^24,25.^

As an early approach to investigate the mechanisms linking the gut microbes to the brain reward system, we showed a highly significant positive correlation between the abundance of the genus *Parabacteroides* and the quantity of HFHS diet ingested during the food preference test. This suggests that *Parabacteroides* could play a potential role in driving the preference for HFHS rather than for CT diet, and stimulate the intake of food rich in fat and sugar, reflecting a functional food reward system. In genetic-obese mice and diet-induced obese mice, *Parabacteroides distasonis* has already been described as a protective bacterium against weight gain and participates in restoring obesity-associated metabolic disorders such as hepatic steatosis and insulin resistance.^26^

We used HFHS during the food preference test because sugar and fat follow different gut-to-brain pathways and have supra-additive effects on the striatal dopamine release.^7^ On the one hand, Han et al. demonstrated that fat uses a PPARα-dependent pathway and the vagus nerve to inform the striatum and induce dopamine release.^27^ On the other hand, sugar-induced striatal dopamine release is mainly due to the metabolism of the sugar in the gut, completed with the information from a portal vein sensor.^28^ To go deeper into the mechanisms, Kaelberer et al. have demonstrated that intestinal enteroendocrine cells are able to communicate with the vagus nerve, in order to transduce an afferent message to the brain and stimulate the reward system.^29^ Since our team has already shown the key interplay between the gut microbiota and enteroendocrine cells to release peptides regulating the food intake (GLP-1, PYY), the investigation of the role of these hormones as key mediators in the crosstalk between the gut microbiota and the reward system, would be an interesting perspective.^30^

**Figure 7.**
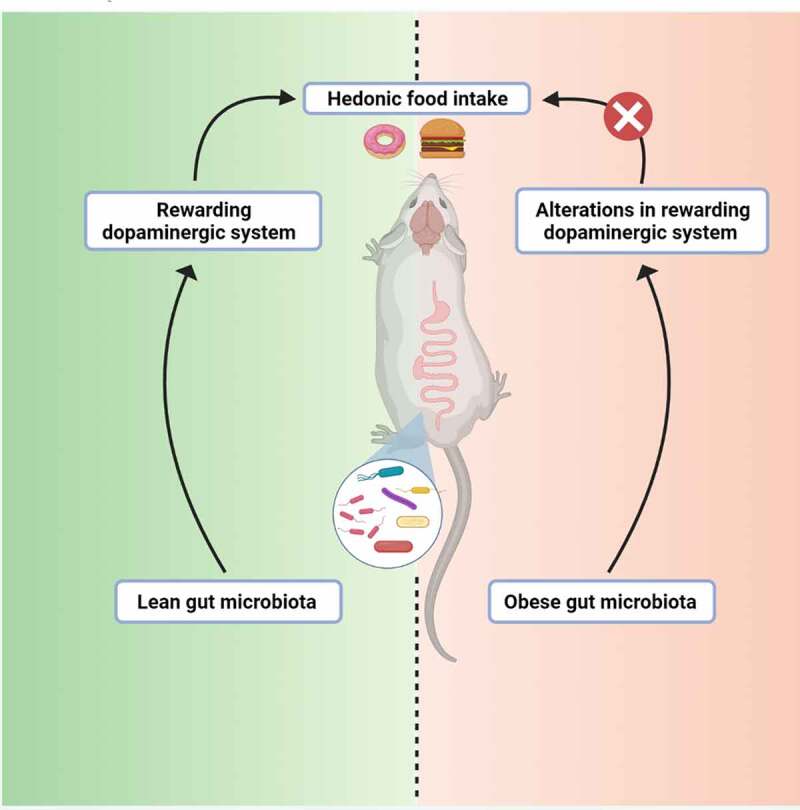
This study demonstrates for the first time the implication of the gut microbiota in the regulation of the reward pathway and hedonic aspects of food intake in mice. The data obtained also reveal the causal role of gut microbiota modifications associated with obesity into the dysregulations of the dopaminergic reward system and the hedonic food intake during obesity

In conclusion, these results demonstrate for the first time the implications of gut microbiota in the regulation of the reward pathway and hedonic aspects of food intake in mice (Figure 7). Our data also reveal the causal role of gut microbiota modifications associated with obesity into the dysregulations of the dopaminergic reward system and the hedonic food intake during obesity (Figure 7). Therefore, we provide here evidence that the gut microbiota could be an interesting therapeutic target to tackle hedonic food intake alterations related to obesity.

## Materials and methods

### Mice and experimental design

All mouse experiments were approved by the ethical committee for animal care of the Health Sector of the UCLouvain, Université catholique de Louvain under the specific number 2017/UCL/MD/005 and performed in accordance with the guidelines of the local ethics committee and in accordance with the Belgian Law of May 29, 2013 regarding the protection of laboratory animals (agreement number LA1230314).

### Donor mice

A cohort of 8-week-old specific-opportunistic and pathogen-free (SOPF) male C57BL/6 J mice (10 mice, n = 5 per group) (Janvier laboratories, France) were housed in a controlled environment (room temperature of 22 ± 2°C,12 h daylight cycle) in groups of two mice per cage, with free access to sterile food (irradiated) and sterile water. Upon delivery, the mice underwent an acclimatization period of 1 week, during which they were fed a control diet (CT, AIN93Mi, Research Diet, New Brunswick, NJ, USA). Then, mice were randomly divided in two groups, and were fed for 5 weeks with a controlled low-fat diet (CT, AIN93Mi) or a high-fat diet (HFD, 60% fat and 20% carbohydrates (kcal/100 g) D12492i, Research diet, New Brunswick, NJ, USA). Body weight, food, and water intake were recorded once a week. The body composition was assessed by using 7.5 MHz time domain-nuclear magnetic resonance (TD-NMR, LF50 Minispec, Bruker, Rheinstetten, Germany). After 4 weeks of follow-up, the mice entered the metabolic chambers to perform the food preference test.

### Recipient mice

A cohort of 3-week-old specific-opportunistic and pathogen-free (SOPF) male C57BL/6 J mice (15 mice, n = 7–8 per group) (Janvier laboratories, France) were housed in a controlled environment (room temperature of 22 ± 2°C,12 h daylight cycle) in groups of two mice per cage, with free access to sterile food (irradiated) and sterile water. Mice were fed a low-fat control diet (CT, AIN93Mi) during the entire transplantation protocol as well as after gut microbiota transplantation. Body weight, food, and water intake were recorded once a week. The body composition was assessed by using 7.5 MHz time domain-nuclear magnetic resonance (TD-NMR, LF50 Minispec, Bruker, Rheinstetten, Germany). After 12 weeks of follow-up, the mice entered the metabolic chambers to assess precisely their food intake and metabolism then perform the food preference test.

### Fecal microbiota transplantation

At the end of the donor experiment, the cecal content was collected in sterile containers and immediately diluted (1:50 w/vol) in sterile Ringer buffer (4,5 g NaCl, 200 mg KCl, 125 mg CaCl_2_). This suspension was then diluted (1:1 v/v) in 20% (w/v) skim milk (Nonfat dry milk, Biorad, 2005668 A) before storage at −80°C. Two CT-fed mice and two HFD-fed mice from the donor cohort were selected as fecal microbiota donors for seven or eight recipient mice per group, respectively, with one donor for three or four recipient mice. As previously described, prior to gut microbiota inoculation, 3-week-old SOPF recipient mice were depleted in intestinal microbiota by daily gavage of a broad-spectrum, poorly absorbed mix of antibiotics during 5 days (100 mg/kg of ampicillin, neomycin, and metronidazole, and 50 mg/kg of vancomycin diluted in sterile water) added with antifungal (amphotericin B 1 mg/kg).^31,32^ Antibiotic treatment was then followed by a bowel cleansing with the administration of 600 µl of PEG solution (PEG/Macrogol 4000, Colofort, Ipsen, France) by oral gavage in two times at 30 min intervals after a 2-h fasting. Colonization was then achieved by intragastric gavage with 300 µl of inoculum three times a week for 1 week. During antibiotic treatment and inoculation, mice were transferred into clean cages 4 times a week. All the recipient mice were kept under CT diet (CT, AIN93Mi).

### Antibiotic-treated mice

A cohort of 8-week-old SOPF male C57BL/6 J mice (40 mice, n = 10 per group) (Janvier Laboratories, France) were housed in a controlled environment (room temperature of 22 ± 2°C,12 h daylight cycle) in groups of two mice per cage, with free access to sterile food (irradiated) and sterile water. Upon delivery, the mice underwent an acclimatization period of 1 week, during which they were fed a control diet (CT, AIN93Mi). Then, mice were fed with a low-fat control diet (CT, AIN93Mi) or a high-fat diet (HFD, D12492i). Treatment continued for 5 weeks, after which the mice entered the metabolic chambers to perform the food preference test. Body weight, food, and water intake were recorded once a week. The body composition was assessed by using 7.5 MHz time domain-nuclear magnetic resonance (TD-NMR, LF50 Minispec, Bruker, Rheinstetten, Germany). The same antibiotic mixture as previously described was administered to half of the mice under the CT diet (Lean_AB) and to half of the mice under HFD diet (DIO_AB) from 7 days prior to the food preference test until the end of the experiment. Amphotericin B treatment ended after 5 days in order to avoid any toxicity.

### Metabolic chambers

After 11 weeks of follow-up, recipient mice were separated and housed individually one week before entering metabolic chambers (Labmaster, TSE systems GmbH, Bad Homburg, Germany). Then, they underwent 4 days of metabolic assessment before the food preference test. The mice were analyzed for oxygen consumption, and carbon dioxide production using indirect calorimetry (Labmaster, TSE systems GmbH). These parameters were expressed as a function of the whole-body weight. Locomotor activity was recorded using an infrared light beam-based locomotion monitoring system (expressed as beam breaks count per hour). Sensors recorded the precise food intake of each diet every 15 minutes. Inside the chambers, measurements were taken every 15 minutes. The final data representation (total, day, or night) corresponds to all the values measured and summed (light phase or dark phase). The means (n = 7) were finally compared between groups.

### Food preference test

During 3 hours in the daylight, mice were exposed to two kinds of diets: a low-fat, control diet (CT, AIN93Mi, Research diet, New Brunswick, NJ, USA) or a high-fat high-sucrose diet (HFHS, 45% fat and 27.8% sucrose (kcal/100 g) D17110301i, Research diet, New Brunswick, NJ, USA) in metabolic chambers (Labmaster/Phenomaster, TSE systems, Germany). Sensors recorded the precise food intake of each diet every 15 minutes.

### Tissue sampling

At the end of each experiment, mice were fed and exposed for 1 h to HFHS before anesthesia with isoflurane (Forene, Abbott, England). This aims to mimic the conditions of the food preference test and stimulate the dopaminergic food reward system. Then the mice were euthanatized by exsanguination and cervical dislocation. Striatum, nucleus accumbens, prefrontal cortex, and caudate putamen were precisely dissected, the cecal content was harvested and immediately immersed into liquid nitrogen, then stored at −80°C for further analysis.

### RNA preparation and real-time qPCR analysis

Total RNA was prepared from the striatum, nucleus accumbens, prefrontal cortex and caudate putamen using TriPure reagent (Roche). Quantification and integrity analysis of the total RNA were performed by running 2 μl of each sample on an Agilent 2100 Bioanalyzer (Agilent RNA 6000 Nano Kit, Agilent). If the RNA integrity number (RIN) obtained less than 6, the sample was excluded from further analyses. cDNA was prepared by reverse transcription of 1 µg total RNA using the GoScript Reverse Transcriptase kit (Promega, Madison, WI, USA). Real-time PCR was performed with the QuantStudio 3 real-time PCR system (Thermo Fisher, Waltham, MA, USA). *Rpl19* RNA was chosen as the housekeeping gene. All samples were performed in duplicate, and data were analyzed according to the 2^−ΔΔCT^ method. The identity and purity of the amplified product were assessed by melting curve analysis at the end of amplification. Sequences of the primers used for real-time qPCR are available in Table 1.

**Table 1.** qPCR primer sequences for the targeted mouse genes

Gene	Forward primer sequence (5’-3’)	Reverse primer sequence (5’-3’)
***RPL19***	GAA-GGT-CAA-AGG-GAA-TGT-GTT-CA	CCT-TGT-CTG-CCT-TCA-GCT-TGT
***D2R***	CCA-AGA-ACG-TGA-GGG-CTA-AG	TGA-GGA-TGC-GAA-AGG-AGA-AG
***D1R***	GAG-CCA-ACC-TGA-AGA-CAC-C	TGA-CAG-CAT-CTC-CAT-TTC-CAG
***TH***	GCC-AAG-GAC-AAG-CTC-AGG-AAC	ATC-AAT-GGC-CAG-GGT-GTA-CG
***DAT***	AAA-TGC-TCC-GTG-GGA-CCA-ATG	GTC-TCC-CGC-TCT-TGA-ACC-TC

### DNA isolation from mouse cecal samples and sequencing

The cecal contents were collected and kept frozen at −80°C until use. Metagenomic DNA was extracted from the cecal content using a QIAamp DNA Stool Mini Kit (Qiagen, Hilden, Germany) according to the manufacturer’s instructions with modifications.^33^ The V1–V3 region of the 16S rRNA gene was amplified from the cecal microbiota of the mice using the following universal eubacterial primers: 27Fmod (5ʹ-AGRGTTTGATCMTGGCTCAG-3ʹ) and 519Rmodbio (5ʹ-GTNTTACNGCGGCKGCTG-3ʹ). Purified amplicons were sequenced utilizing a MiSeq following the manufacturer’s guidelines. Sequencing was performed at MR DNA (www.mrdnalab.com, Shallowater, TX, USA). Sequences were demultiplexed and processed using the QIIME pipeline (v1.9 using default options: Q25, minimum sequence length = 200 bp, maximum sequence length = 1000 bp, maximum number of ambiguous bases = 6, maximum number of homopolymers = 6, maximum number of primer mismatches = 0). For the 22 samples analyzed, 102 OTUs have been identified (97% similarity). The minimum number of sequences per sample was 48 170 and the maximum number of sequences per sample was 86 360. The median number of sequences per sample was 61 143 and the mean number of sequences per sample was 63 7392 ± 10 798 (standard deviation). The Q25 sequence data derived from the sequencing process were analyzed with the QIIME 1.9 pipeline. Briefly, the sequences were depleted of barcodes and primers. Sequences of 1000 bp were then removed; sequences with ambiguous base calls and with homopolymer runs exceeding 6 bp were also removed. Sequences were denoised, and operational taxonomic units (OTUs) were generated. Chimeras were also removed. OTUs were defined by clustering at 3% divergence (97% similarity). The final OTUs were taxonomically classified using BLASTn against a curated Greengenes database. PCoA was generated with QIIME using the unweighted UniFrac distance matrix between the samples and as previously described.^34–37^ The data for this study have been deposited in the European Nucleotide Archive (ENA) at EMBL-EBI under accession number PRJEB46582.

### Statistical analysis

Statistical analyses were performed using GraphPad Prism version 8.1.2 for Windows (GraphPad Software, San Diego, CA, USA) except for microbiota analyses as described above. Data are expressed as mean ± SEM. Differences between the two groups were assessed using unpaired Student’s t-test. In case variance differed significantly between groups according to the Fisher test, a non-parametric (Mann–Whitney) test was performed. Differences between more than two groups were assessed using one-way ANOVA or two-way ANOVA if repeated measurements, followed by Tuckey or Bonferroni, respectively, post-hoc test. If variance differed significantly between groups, a non-parametric Kruskal–Wallis test was performed, followed by the Dunnett post-hoc test.

## Supplementary Material

Supplemental Material
