# Supplementary figures and images for: Gut microbes participate in food preference alterations during obesity
Source: Gut Microbes. 2021 Aug 23;13(1):1959242. doi: 10.1080/19490976.2021.1959242 (PMC8386729; doi:10.1080/19490976.2021.1959242)

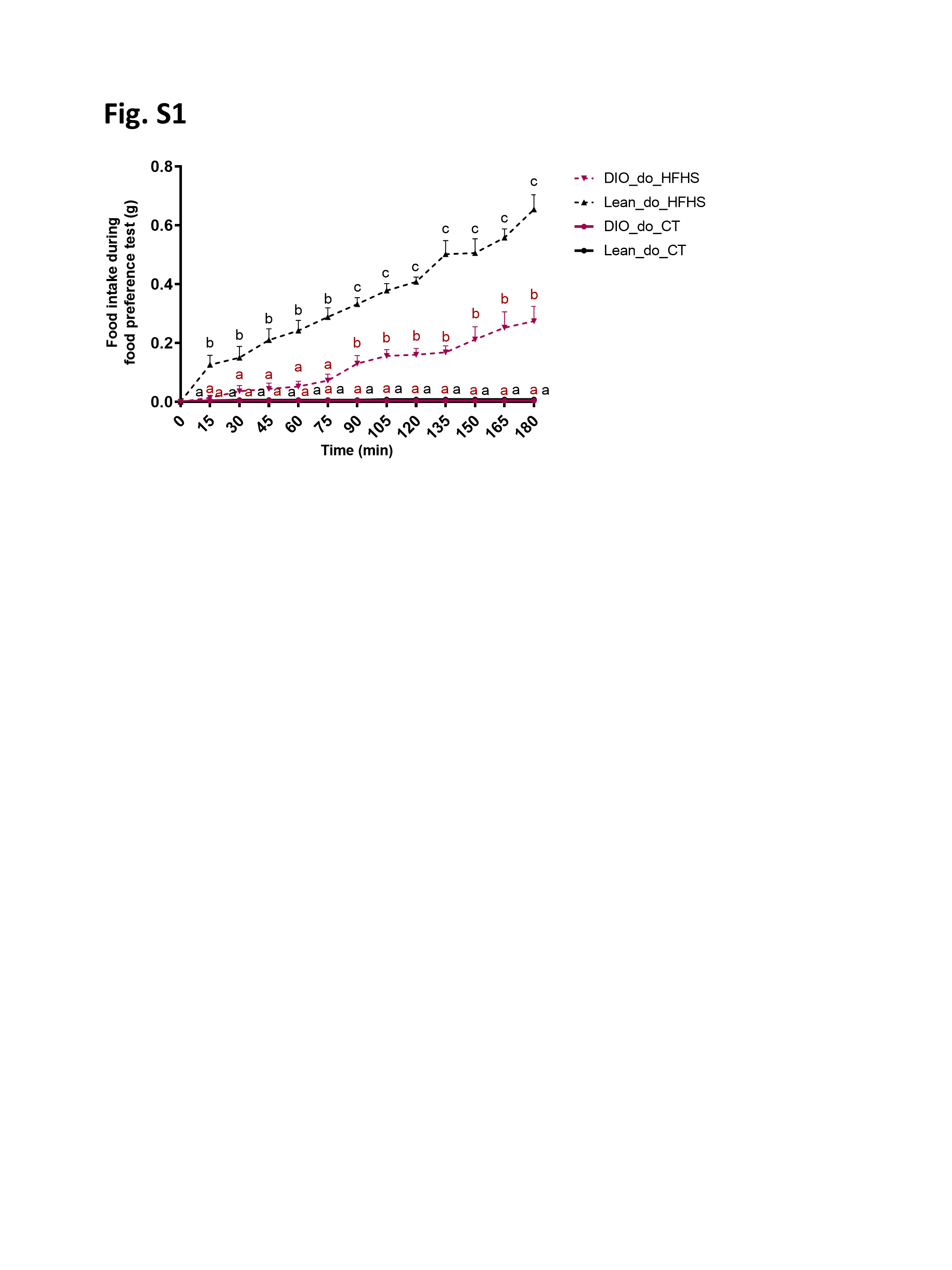

Supplement: Supplemental Material [file KGMI_A_1959242_SM7898.zip › suppl/Fig S1 revision.tif]

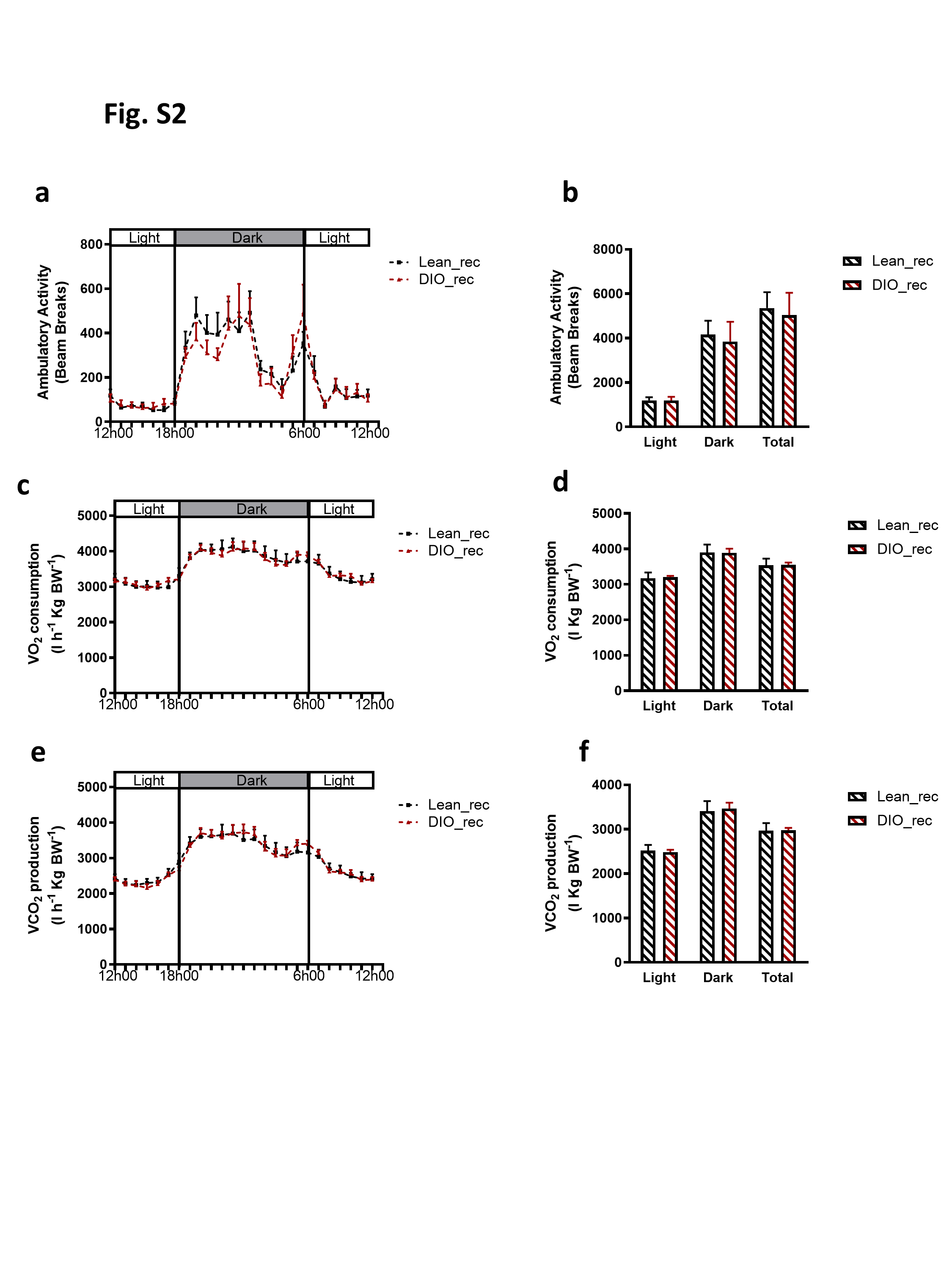

Supplement: Supplemental Material [file KGMI_A_1959242_SM7898.zip › suppl/Fig S2 revision.tif]

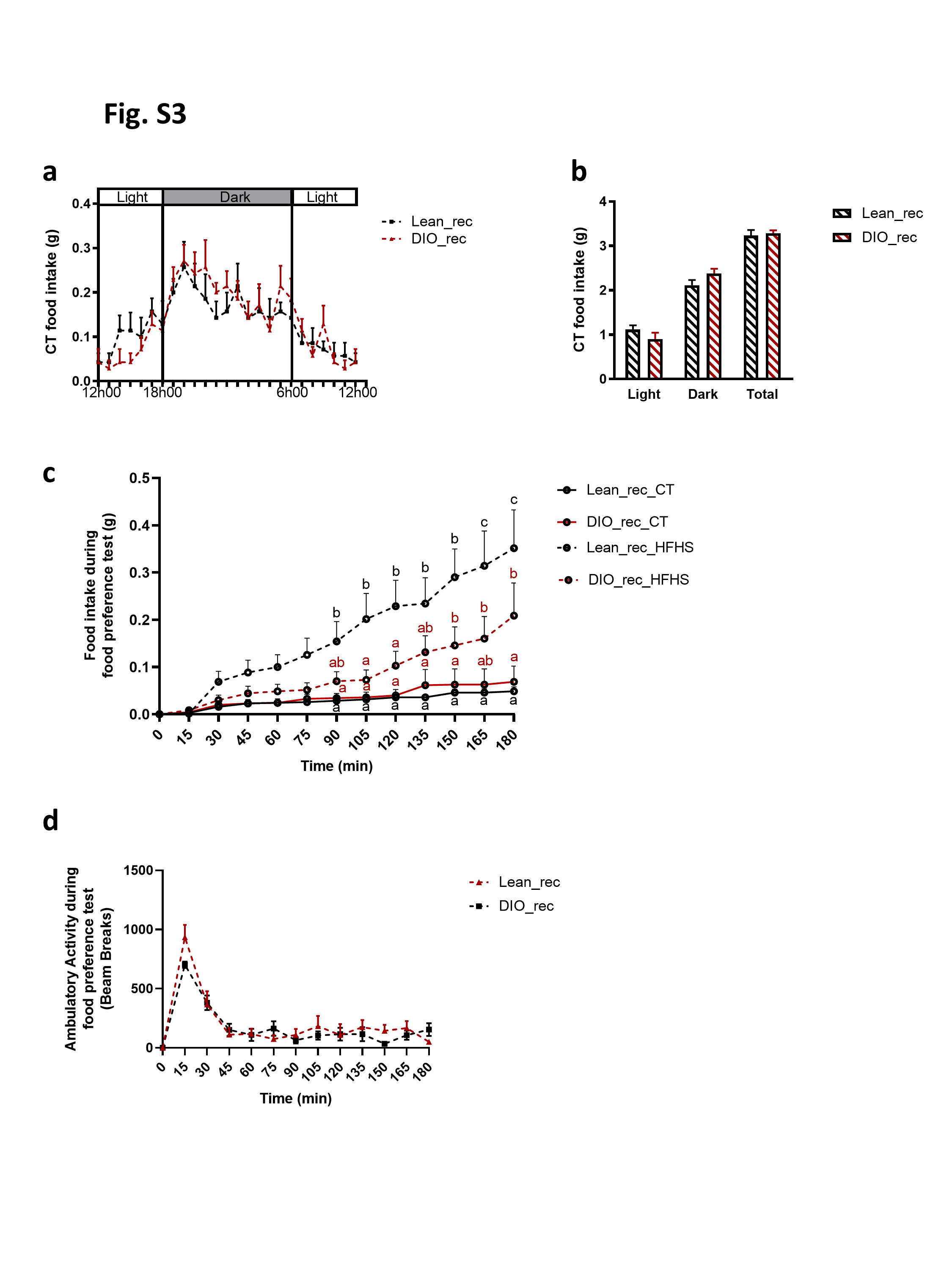

Supplement: Supplemental Material [file KGMI_A_1959242_SM7898.zip › suppl/Fig S3 revision.tif]

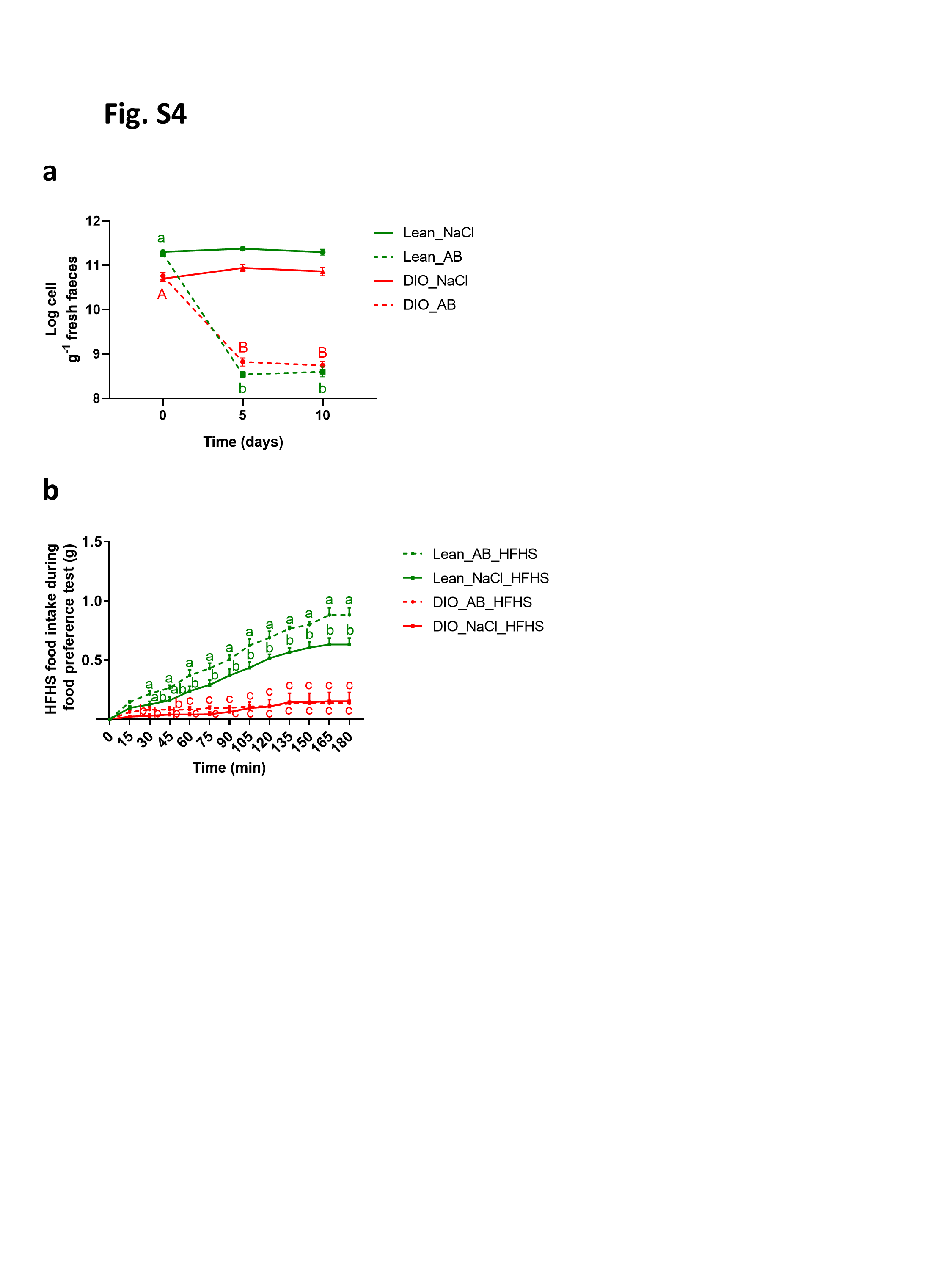

Supplement: Supplemental Material [file KGMI_A_1959242_SM7898.zip › suppl/Fig S4 revision.tif]

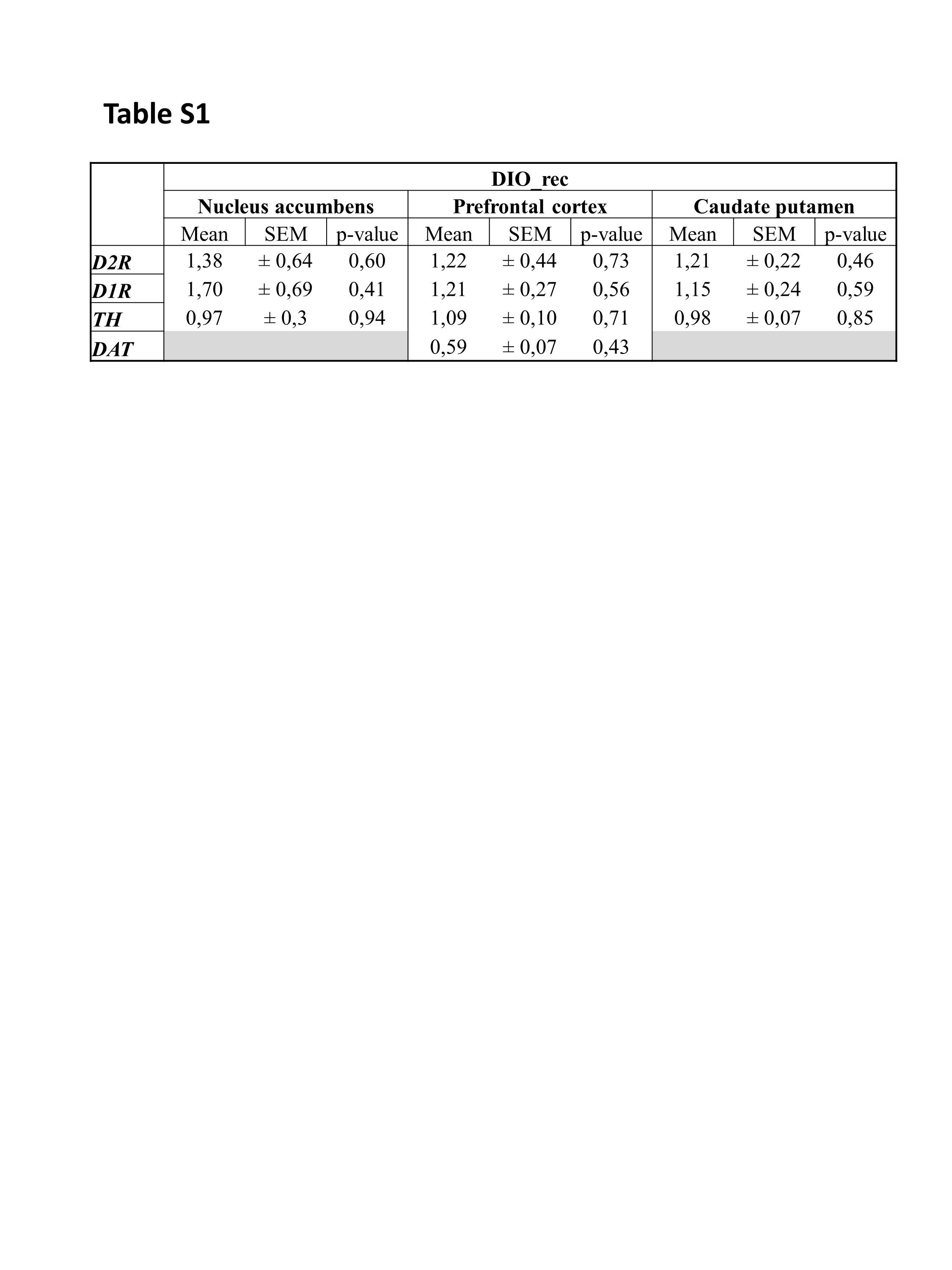

Supplement: Supplemental Material [file KGMI_A_1959242_SM7898.zip › suppl/Table S1 revision.tif]
